# Analysis of biodiesel by high performance liquid chromatography using refractive index detector

**DOI:** 10.1016/j.mex.2017.07.002

**Published:** 2017-07-19

**Authors:** Mahin Basha Syed

**Affiliations:** a Biochemistry Lab, P.M. Sayeed Calicut University Centre, Androth, U.T. of Lakshadweep, 682551, India; b Department of Chemical Engineering, Annamalai University, Annamalai Nagar, 608002, Tamil Nadu, India

**Keywords:** HPLC, Biodiesel, Refractive index, UV detector

## Abstract

High-performance liquid chromatography (HPLC) was used for the determination of compounds occurring during the production of biodiesel from karanja and jatropha oil. Methanol was used for fast monitoring of conversion of karanja and jatropha oil triacylglycerols to fatty acid methyl esters and for quantitation of residual triacylglycerols (TGs), in the final biodiesel product. The individual sample compounds were identified using HPLC. Analysis of fatty acid methyl esters (FAMES) in blends of biodiesel by HPLC using a refractive index and a UV detector at 238 nm. Individual triacylglycerols, diacylglycerols, monoacylglycerols and methyl esters of oleic, linoleic and linolenic acids and free fatty acids were separated within 40 min. Hence HPLC was found to be best for the analysis of biodiesel. Analysis of biodiesel by HPLC using RID detector. Estimation of amount of FAMES in biodiesel. Individual triacylglycerols, diacylglycerols, monoacylglycerols and methyl esters of oleic, linoleic and linolenic acids and free fatty acids were separated within 40 min.

## Graphical abstract

**Figure:**
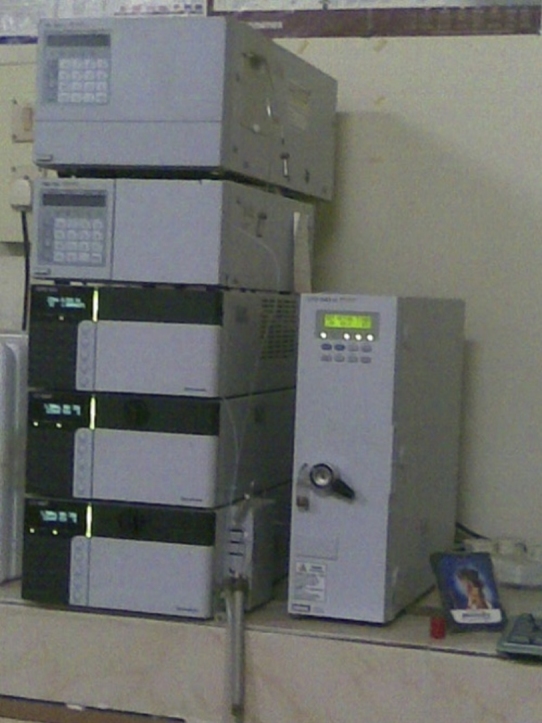

## Materials

The standards (mixture of methyl esters) were purchased from Sigma-Aldrich Chemicals Pvt. Ltd, India.

## Method details

### Jatropha and Karanja oil characterisitics

The non-edible crude jatropha and karanja oils were purchased from local market and stored at 4 °C to avoid rancidity of the vegetable oil. Major fatty acid profiles of jatropha and karanja oils were given in Table 1. The characteristics of jatropha and karanja oils were determined according to the standard methods given in Table 1.

**Table 1:** Fatty acid profile of Jatropha and Karanja oil.

Fatty acids with (carbon atoms:double bonds)	Composition of Karanja oil (w/w of oil)	Composition of Jatropha oil (w/w of oil)
Myristic acid (14:0)	–	1.4
Palmitic acid (16:0)	10.6	15.6
Stearic acid (18:0)	6.8	9.7
Oleic acid (18:1)	49.4	40.8
Linoleic acid (18:2)	19.0	32.1
Arachidic acid (20:0)	4.1	0.4
Gadoleic acid (20:1)	2.4	–
Behenic acid (22:0)	5.3	–
Lignoceric acid (24:0)	2.4	–

### Lipase enzyme – biocatalyst

The following lipases were procured from Sigma – Aldrich Chemicals Pvt. Ltd., Bangalore: and other chemicals were listed in Table 2

• *Pseudomonas fluorescens* lipase (300 U/mg)
• *Candida rugosa* lipase (700 U/mg)
• *Rhizopus arrihizus* lipase (10.5 U/mg)
• *Aspergillus oryzae* lipase (15.5 U/mg)
• *Candida antartica* lipase (600 U/mg)

**Table 2:** Specifications of chemicals employed in the investigation.

S. No	Chemicals	Specifications
1	Potato Dextrose Agar	LR – Himedia, India
2	Methanol	HPLC grade – Merck, India
3	HPLC Water	HPLC grade – Merck, India
4	Ethanol	LR-SD Fine Chemicals Limited, India
5	Oleic acid	LR-SD Fine Chemicals Limited, India
6	Methyl trimethoxylane	LR-SD Fine Chemicals Limited, India
7	n- Hexane	HPLC grade – Nice Chemicals, India
8	n- Butyl Alcohol	HPLC grade – Nice Chemicals, India
9	Petroleum ether	AR – Nice Chemicals, India
10	Propanol	LR – Nice Chemicals, India
11	Toluene	AR – Nice Chemicals, India
12	Isopropyl Alcohol	LR – Nice Chemicals, India
13	n-Heptane	LR – Nice Chemicals, India
14	Orthophosphoric acid	AR – Nice Chemicals, India
15	Iso octane	LR – Nice Chemicals, India
16	T- Butanol	LR – Nice Chemicals, India
17	Amyl alcohol	LR – Nice Chemicals, India
18	Sodium Alginate	LR – Nice Chemicals, India
19	Olive Oil	LR-SD Fine Chemicals Limited, India
20	Polypeptone	LR – Himedia, India
21	Potassium dihydrogen phosphate	AR – Himedia, India
22	Magnesium Sulphate	LR-SD Fine Chemicals Limited, India
23	Sodium nitrite	LR-SD Fine Chemicals Limited, India
24	MSTFA Derivatization Reagent	Sigma Aldrich, USA
25	Standard Fatty acid methyl ester mixture	Sigma Aldrich, USA
26	Standard Triolein, Diolein and Mono olein mixture	Sigma Aldrich, USA

### HPLC analysis for biodiesel

A High Performance Liquid Chromatographic system (HPLC Model- LC 20 AT Prominence, Shimadzu, Japan) fitted with Refractive index detector (RID-10A, Shimadzu, Japan) and millennium 32 system software was used to quantify the fatty acid methyl esters produced during reaction. Separations were carried out on a 238 nm in Luna C_18_ column of particle size 5 mm and (250 × 4.6 mm) I.D. Methanol mobile phase was filtered through a 0.45 μm membrane filter (Millipore), and then degassed ultrasonically prior to use. The flow rate was 1 mL min^−1^, the injection volume was 20 μL and the column oven temperature was maintained at 40 °C. Each component in the samples analyzed was identified by comparing its retention time with that of the respective standards. Quantification was carried out by integration of the peaks using external standards followed by calculating the% yield as weight of methyl esters produced to weight of oil initially taken [1].

### Preparation of standard

**Fig. 1:**
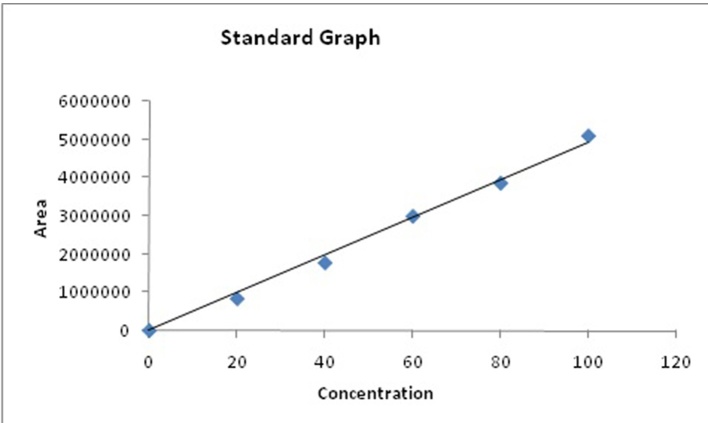
Standard graph of biodiesel using refractive index detector.

A stock solution was prepared by dissolving 40 mg of standard in 2 mL of methanol. Five serial dilutions were made from the stock solution namely 2, 4, 8, 12 and 16 mg/ml using methanol as solvent. The base line for HPLC system was set according to the program mentioned in the analysis section. Once the base line was set, the samples from the stock solution were analyzed by injecting 20 μL and the corresponding peak area values are noted. The particular methyl ester peaks are identified by taking the retention time as reference. The calibration charts were drawn for the values of peak area obtained vs concentration of the sample for each methyl esters individually as shown in Fig. 1.

### Additional information

HPLC was applied for the analysis of biodiesel than GC analysis. LC was found to be operationally superior to GC because of the aforementioned reasons, and it was directly applicable to most biodiesel fuels [2]. Simultaneous determination of aromatic compounds and FAME in blends of biodiesel with petrodiesel by HPLC using a refractive index and a UV detector [3]. It has been used to identify various components of biodiesel mixtures including fatty acid methyl esters, triglycerides, diglycerides, monoglycerides, and fatty acids, among others. HPLC analysis time is generally shorter than GC, and no derivitization step is needed. Additionally, lower analysis temperatures allow for the use of standard columns [4].
